# The effect of maternal chromium status on lipid metabolism in female elderly mice offspring and involved molecular mechanism

**DOI:** 10.1042/BSR20160362

**Published:** 2017-04-28

**Authors:** Qian Zhang, Xiaofang Sun, Xinhua Xiao, Jia Zheng, Ming Li, Miao Yu, Fan Ping, Zhixin Wang, Cuijuan Qi, Tong Wang, Xiaojing Wang

**Affiliations:** 1 Key Laboratory of Endocrinology, Translational Medicine Centre, Ministry of Health, Department of Endocrinology, Peking Union Medical College Hospital, Peking Union Medical College, Chinese Academy of Medical Sciences, Beijing 100730, China; 2 Department of Endocrinology, The Affiliated Hospital of Qingdao University, Qingdao, Shandong 266003, China

**Keywords:** chromium, development, gene expression, lipid metabolism, PPAR pathway

## Abstract

Maternal malnutrition leads to the incidence of metabolic diseases in offspring. The purpose of this project was to examine whether maternal low chromium could disturb normal lipid metabolism in offspring, altering adipose cell differentiation and leading to the incidence of lipid metabolism diseases, including metabolic syndrome and obesity. Female C57BL mice were given a control diet (CD) or a low chromium diet (LCD) during the gestational and lactation periods. After weaning, offspring was fed with CD or LCD. The female offspring were assessed at 32 weeks of age. Fresh adipose samples from CD–CD group and LCD–CD group were collected. Genome mRNA were analysed using Affymetrix GeneChip Mouse Gene 2.0 ST Whole Transcript-based array. Differentially expressed genes (DEGs) were analysed based on gene ontology (GO) and Kyoto Encyclopedia of Genes and Genomes (KEGG) pathway analysis database. Maternal low chromium irreversibly increased offspring body weight, fat-pad weight, serum triglyceride (TG) and TNF-α. Eighty five genes increased and 109 genes reduced in the offspring adipose of the maternal low chromium group. According to KEGG pathway and String analyses, the PPAR signalling pathway may be the key controlled pathway related to the effect of maternal low chromium on female offspring. Maternal chromium status have long-term effects of lipid metabolism in female mice offspring. Normalizing offspring diet can not reverse these effects. The potential underlying mechanisms are the disturbance of the PPAR signalling pathway in adipose tissue.

## Introduction

More and more studies in humans reveal that undernutrition *in utero* leads to the reduction in birth weight [1], long-term modification in metabolic status [2] and is considered as a risk factor for obesity [3,4]. However, most animal models in which mechanism of this relationship has been studied on the lack of macronutrients *in utero* only. In fact, micronutrients, including minerals, have key roles in the organ development, body function and reproduction [5].

Chromium (Cr(III)) is considered as important nutrient in keeping normal lipid metabolism, balancing appetite, inhibiting fat mass and elevating lean body mass [6]. Chromium supplementation can significantly cut down total cholesterol (TC) and low-density lipoprotein (LDL) levels in Type 2 diabetes (T2D) subjects [7–10], women with polycystic ovary syndrome [11] and high fat diet rats [12,13]. The suggested minimum daily intake of chromium is 30 μg. But, the average dietary chromium intake in adults is far below this standard in many areas [14,15]. Particularly, pregnant women and elderly subjects are more prone to chromium deficiency [16,17]. Because they have elevated metabolic stress and inhibited absorption ratio of chromium [18,19]. Serum chromium levels in T2D patients were half of that in control subjects. Inverse correlation of HbA1c and serum chromium concentration was also addressed [17]. Vincent, J.B. [20] reports that dietary chromium deficiency elevates serum TC. This elevation of serum TC can be relieved by chromium supplementation [20]. Long term maternal chromium insufficiency increased body fat in WNIN rat offspring and that is probably due to increased oxidative stress [21]. Moreover, the up-regulation of 11β-hydroxysteroid dehydrogenase 1 (11β-HSD1) and leptin may contribute to the elevated adiposity in these offspring.

Previous studies on chronic modification in gene expression affected by maternal low chromium usually performed a candidate gene approach, whereas this method does not assess the specificity of the modifications led by chromium deficiency in the transcriptome level of the offspring and gene networks in lipid metabolism [21]. In the present study, we have adopted a genome-wide microarray to assess the influence by maternal chromium nutrition deficiency during pregnancy on gene expression in offspring.

We hypothesized that there are key genes and molecular pathways which express differently in offspring adipose when dams are fed with different chromium diets. To induce this effect, pregnant dams were given either a control diet (CD) or a low chromium diet (LCD) from gestational day 0. We analysed 32-week old offspring adipose whole genomic expression to look for the key genes and pathways involved with metabolic disturbance from maternal LCD.

## Materials and methods

### Animals and diets

All experiments related to animals were followed with the Approval of the Animal Care Committee of the Peking Union Medical Hospital (permit number: MC-07-6004) and were performed strictly following the standards of the Animal Ethics Committee of the Peking Union Medical Hospital. We tried our best to minimize animal pain. Seven-week-old female and male C57BL mice (17.8 ± 1.5 g) were obtained from Institute of Laboratory Animal Science, Chinese Academy of Medical Sciences and Peking Union Medical College (Beijing, China, SCXK-2013-0107). Mice were mated at the onset of pro-oestrous. The presence of sperm in the vaginal smear is considered as impregnation. Pregnant mice (*n*=16) were caged individually and kept at 22°C on a 12 h light and 12 h dark cycle. From the first day of gestation, the mice were given diets with different chromium concentration. However, all the other contents of diet were the same (*n*=8/group). The CD (a casein-based diet based on the American Institute of Nutrition AIN-93G diet, CD), contained 1.19 mg chromium/kg diet and the low-chromium diet (only excluded in chromium, LCD) contained 0.14 mg chromium/kg diet. Atomic absorption spectrometer (TAS986, Beijing Persee General Corporation, Beijing, China) was used to assess the content of chromium in the diet. All diets were obtained from Research Diets (New Brunswick, NJ, U.S.A.). In the present study, low-chromium diet was just excluded 90% chromium potassium sulfate from CD.

**Figure 1:**
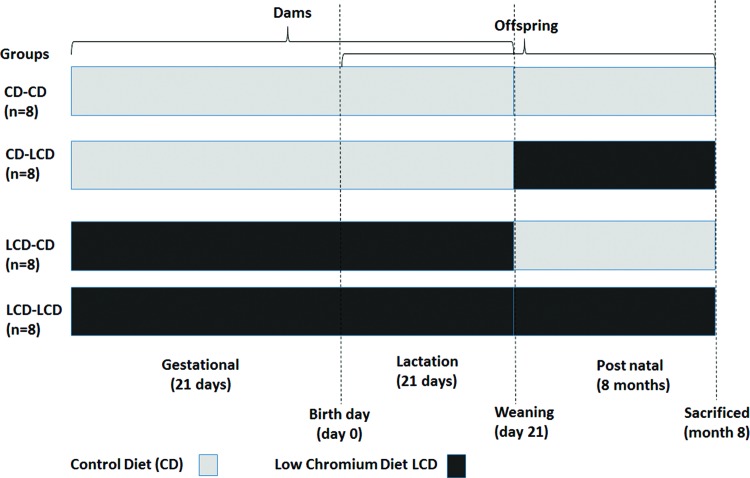
Timeline of animal experiment Time schedule of the animal experiment. Female mice received the CD or LCD during the gestational and lactation periods. The female offspring were monitored at birth, 3 weeks and 8 months of age.

The litter size in LCD group is less than that in CD group (6.00 ± 0.76 compared with 8.50 ± 0.53, *P*<0.05). On the first day of the new born, the litter size in mother was randomly modified to six pup mice (three males, three females, if possible) to ensure adequate and same nutrition until the pup mice were weaned. At weaning, blood was gained from the supraorbital sinus of the mothers, after fasting overnight, to assess serum chromium levels. Pups were weaned at week 3 of lactation and remained on the diet type or transferred to another diet (Figure 1, *n*=8/group, one female pup from each litter was randomly assigned to the experimental groups). Food and water were provided freely. The present study focused on female offspring due to the varying phenotypic effects of maternal unbalanced nutrition on male and female offspring [22]. At 32 weeks of age, female mice (*n*=8 per group) were killed. The blood sample was obtained from the intraorbital retrobulbar plexus after 10 h fasting and anaesthetized (ketamine 100 mg/kg i.p. Pharmacia and Upjohn Ltd, Crawley, U.K.). The adipose tissue of the offspring was quickly colledted and kept at –80°C for further analysis.

### Measurement of serum chromium level

Serum was obtained from the dams at weaning and from the offspring at 8 months of age for chromium analysis. The serum chromium concentration was measured by atomic absorption spectrometer (Atomic Absorption Spectrophotometer, Hitachi, Japan).

### Measurement of body weight and food consumption

Body weight was assessed at weaning for dams and at birth (day 0), weaning (day 21), and at 8 months for offspring. Food consumption was assessed at 8 months for the offspring. Food consumption was determined for each group by weighing the total amount of food given at the start of the week and then subtracting the amount of food remaining at the end of the week. The average food consumed per mouse was then obtained by dividing by the number of mice.

### Measurement of adiposity index

The adiposity index (AI) and the index of visceral adiposity, were computed according to Taylor and Philips [23]. For this purpose, retroperitoneal, mesenteric and ovarian fat pads were quickly excised from the offspring at the time of their killing, their fresh weights were determined and the AI was computed as equation:

$$\text{AI }=\frac{\text{Sum of the fresh weights of the three fat deposits}}{\text{Body weight}}\times 100$$

### Assays of biochemical parameters in offspring

Blood samples were obtained from 8-months-old offspring. The serum leptin, adiponectin, TNF-α, IL-6 and IL-1β concentrations were measured using a commercially available ELISA kit (Abcam, Cambridge, MA, U.S.A.). All samples were analysed in duplicate and the intra-assay coefficient of variation in leptin, adiponectin, TNF-α, IL-6 and IL-1β was 4.7, 4.9, 5.3, 4.7 and 4.5% respectively. Serum TC, triglyceride (TG), HDL-C and LDL-C concentrations were determined by an enzyme end-point method using a commercial kit (Roche Diagnostics, GmbH, Mannheim, Germany).

### mRNA preparation, labelling and hybridization

Total adipose tissue RNA was extracted using a total RNA Isolation Kit (mirVana™, Ambion, Sao Paulo, SP, Brazil). Six microarrays were performed from the CD–CD group (*n=*3) and LCD–CD group (*n=*3). Briefly, 300 μg of total RNA was reverse transcribed into cDNA. Samples were then transcribed into cRNA and labelled with the fluorescent dye Cy3 (for test sample) or Cy 5 (for reference sample). Then, the samples were hybridized to an Affymetrix GeneChip Mouse Gene 2.0 ST whole transcript-based array (Affymetrix, Santa Clara, CA, U.S.A.). This gene array includes 26515 genes. The slides were then scanned by Affymetrix GeneChip Command Console software (Affymetrix, Santa Clara, CA, U.S.A.). All data were transferred into GeneSpring version 12.5 (Agilent Technologies, Palo Alto, CA, U.S.A.) to normalize the data, control the quality and analyse. Fold change and *P* value (from *t* test) were used to identify differentially expressed genes (DEGs). The threshold was set at a fold change ≥1.5 and a *P* value ≤0.05.

### Pathway and network analysis

We conducted hierarchical clustering for DEGs in the LCD–CD group compared with the CD–CD group. A heat map was constructed by compiling the DEG into TIGR MeV (MultiExperiment Viewer) software (http://www.tm4.org/) [24]. To fully clarify the biological meaning of the cluster of DEGs, gene ontology (GO) classification system and Kyoto Encyclopedia of Genes and Genomes (KEGG) pathways were analysed by DAVID (database for annotation, visualization and integrated discovery) software (http://david.abcc.ncifcrf.gov/) [25]. String software was used to draw the genetic interaction network (http://string-db.org/) [26].

### Real-time PCR validation

For the analysis of the expression level of zinc finger protein (ZFP) 423 (*Zfp423*), lipoprotein lipase (*Lpl*), fatty acid binding protein 3 (*Fabp3*), sterol regulatory element binding transcription factor 1 (*Srebf1*), peroxisome proliferator activated receptor γ (*Pparg*) and CCAAT/enhancer-binding protein (*Cebpa*) in the CD–CD group and the LCD–CD group, real-time PCR was performed by using SYBR Green Master Mix. Specific primers for each gene were list in Table 1. Reactions were perfomed in a total volume of 20 μl, including 5 μl cDNA (diluted 1:100), 10 μl SYBR Green Master Mix (Applied Biosystems, Foster City, CA, U.S.A.) and 2.5 μl of each specific primer (5 nM) and run in the ABI Prism 7500 real-time PCR system (Applied Biosystems, Foster City, CA, U.S.A.). The cycling conditions were set at 95°C for 10 min; 45 cycles at 95°C for 15 s and 60°C for 1 min. *Gadph* is measured for normalization. The ΔΔ*C*_t_ method was calculated to assess the relative quantifications [27].

**Table 1:** Oligonucleotide sequences for qPCR analysis

Gene symbol	Genebank ID	Forward primer	Reverse primer	Product size
*Zfp423*	NM_033327	GATGTGATTGCTTGGCTAT	ACCGATTATATTCATTACAGAGT	123
*Lpl*	NM_008509	AGTCTGTTGTGGTTATCTG	GTTAAGTTGGCTCAGTGA	92
*Fabp3*	NM_010174	AAGCCTACTACCATCATC	GATCTCTGTGTTCTTGAAG	78
*Srebf1*	NM_011480	CTGTTGTCTACCATAAGC	TTAGTGCCAGGTTAGAAG	84
*Pparg*	NM_001127330	GCATCAGGCTTCCACTAT	GCATCAGGCTTCCACTAT	75
*Cebpa*	NM_007678	AGGAACTTGAAGCACAAT	ACACAGAGACCAGATACA	109

### Statistical analysis

All data are shown as mean ± S.D. ANOVA, followed by Tukey’s post hoc test and unpaired Student’s *t* test were used to analyse the data. Fisher’s exact test was used to perform GO and KEGG pathway analysis. *P*<0.05 was considered significant. All analysis were done with GraphPad Prism Software version 5.0 (San Diego, CA, U.S.A.).

## Results

### Effect of LCD on body weight and serum chromium concentration in dams

Serum chromium was lower in LCD (0.45 ± 0.09 ng/ml) than in CD (0.89 ± 0.22 ng/ml) mice (*P*<0.01). However, there was no difference between LCD and CD mice in the levels of body weight at weaning (23.1 ± 3.5 compared with 22.3 ± 3.4 g).

### Effect of maternal LCD on serum chromium level in pups

**Figure 2:**
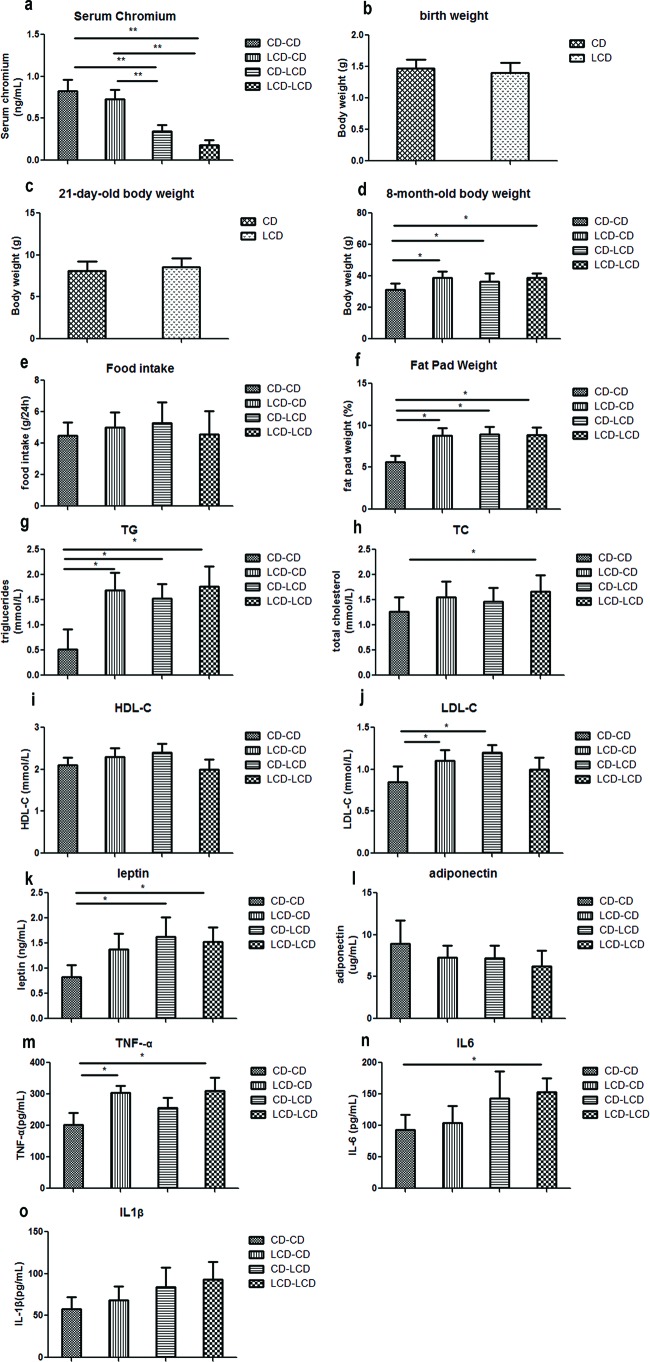
Metabolic indicators affected by maternal low chromium diet Serum chromium level (**a**) at 8 months of age in offspring, birth weight (**b**), weaning weight (**c**), and body weight (**d**), food intake (**e**), fat pad weight (**f**), serum TG (**g**), TC (**h**), HDL-C (**i**), LDL-C (**j**), leptin (**k**), adiponectin (**l**), TNF-α (**m**), IL-6 (**n**) and IL-1β (**o**) at 8 months of age in offspring. Values are mean ± S.D. (*n*=8); *P<0.05, ** *P*<0.01.

At 32-weeks compared with the CD–CD group and LCD–CD group, serum chromium in the CD–LCD group and LCD–LCD group significantly decreased (*P*<0.01). Importantly, the serum chromium level in the LCD–CD group was corrected to normal (Figure 2a).

### Effect of maternal LCD on body weight and food consumption in pups

Despite comparable birth (day 0) weights and weaning (day 21) weights, body weight at 8 months of age in the CD–LCD group, LCD–LCD group and LCD–CD group was higher than in the CD–CD group (*P*<0.05, Figure 2b–d), although food intake was comparable among the groups (Figure 2e).

### Effect of maternal LCD on fat-pad weight in pups

Fat-pad weight in the CD–LCD, LCD–CD and LCD–LCD groups was higher than in the CD–CD group (*P*<0.05, Figure 2f). Interestingly, a reverse to the CD did not normalize the fat-pad weight.

### Effect of maternal LCD on serum lipid profile in pups

The LCD–LCD group offspring had higher serum TC and TG (*P*<0.05, Figure 2g,h). Meanwhile, the CD–LCD group had higher TG and LDL-C (*P*<0.05, Figure 2g,j). Importantly, TG and LDL-C levels in the LCD–CD group did not return to normal (Figure 2g,j). No differences were observed in the serum HDL-C levels among the different groups (Figure 2i).

### Effect of maternal LCD on serum leptin, adiponectin and pre-inflammation cytokines in pups

Serum leptin, TNF-α and IL-6 levels were higher in the LCD–LCD group than in the CD–CD group (*P*<0.05, Figure 2k,m,n). Serum TNF-α level was still higher in the LCD–CD group than the CD–CD group (*P*<0.05, Figure 2m). Serum adiponectin and IL-1β levels were comparable in all four groups (Figure 2l,o).

### Gene expression profile from microarray analysis

**Figure 3:**
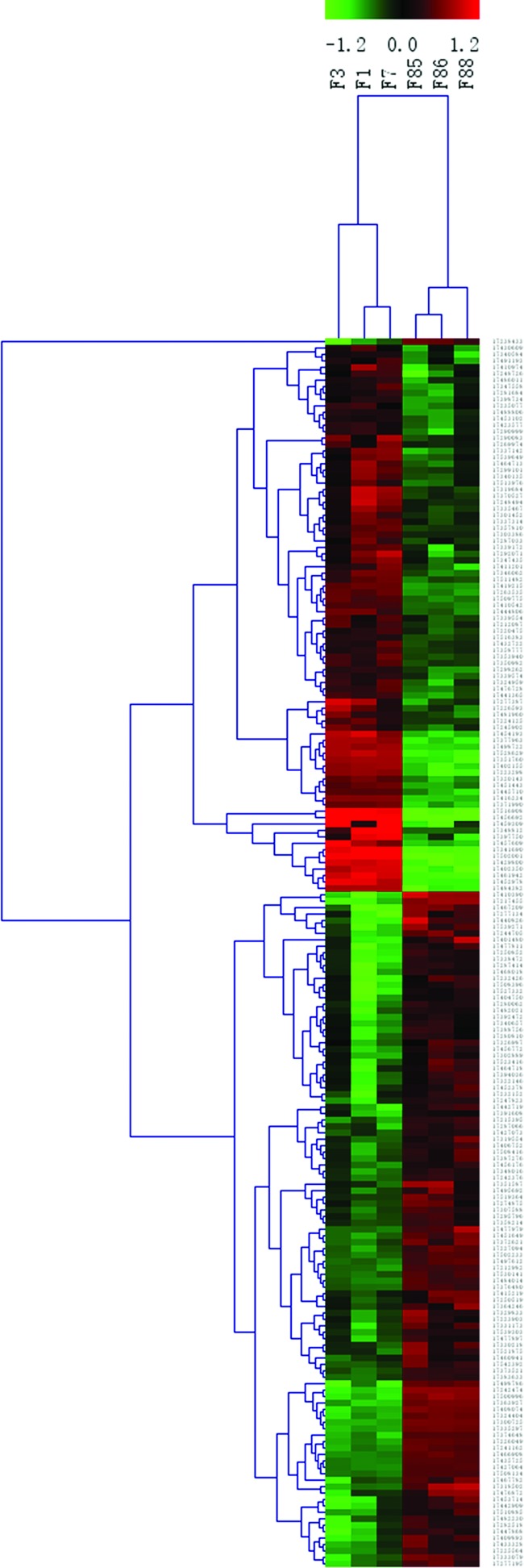
Hierarchical clustering of differently expressed genes between LCD-CD group and CD-CD group Hierarchical clustering of the 1.5-fold up-regulated and down-regulated genes. ‘Red’ indicates high relative expression; ‘green’ low relative expression. Samples F1, F3, F7 belong to LCD–CD group; samples F85, F86 and F88 belong to CD–CD group.

**Figure 4:**
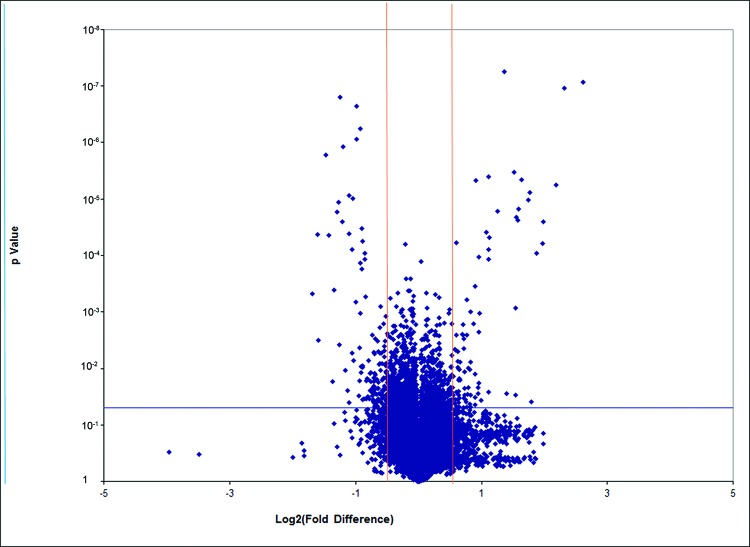
Volcano Plot of all genes in gene array (LCD-CD group vs CD-CD group) Volcano plot of genes in gene array. The red lines represent 1.5-fold up- and down-regulation and the blue line shows a *P*-value of 0.05. The dots in right block above the blue line are up-regulated genes; the dots in left block above the blue line are down-regulated genes.

The microarray analysis was performed from the CD–CD group (three samples) and the LCD–CD group (three samples). Hierarchical clustering analysis of the six array expression data showed a homogeneous expression profile between the samples of the two groups (Figure 3). By setting the threshold for the fold change (FC) to ±1.5 and the *P* value at ≤0.05, compared with those in the CD–CD group, we identified that the expression of 191 genes was significantly changed in the LCD–CD group (85 up-regulated, 109 down-regulated, Figure 4).

### Gene oncology, pathway and network analysis

**Figure 5:**
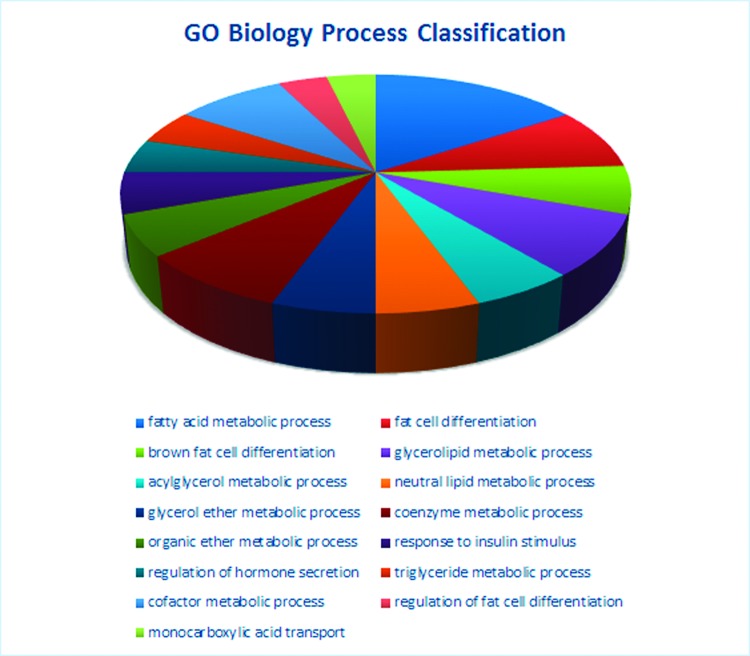
GO biological process classification affected by maternal low chromium diet The most obvious changes in GO biological process classification.

**Figure 6:**
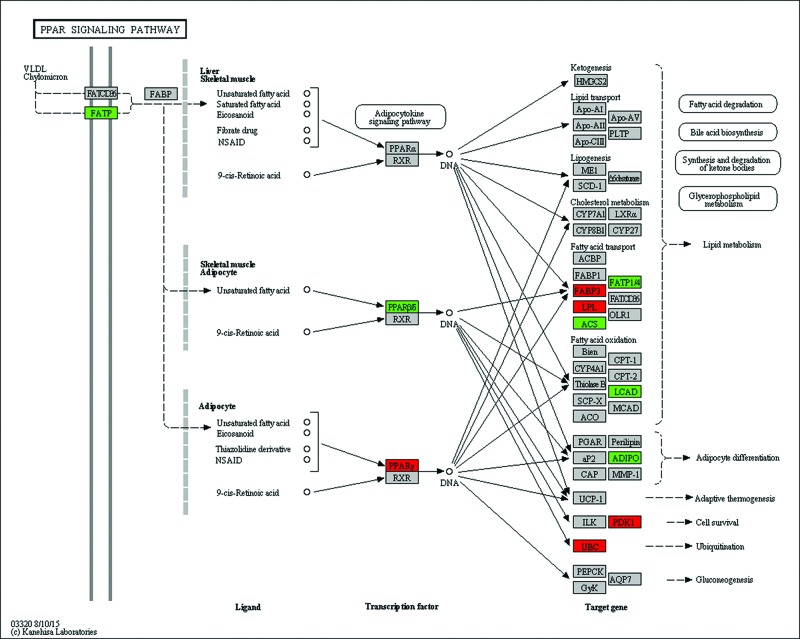
PPAR sinaling pathway affected by maternal low chromium diet All the different expression genes in PPAR pathway. Red represents up-regulated; green represents down-regulated; grey represents no significant change.

We analysed the microarray dataset using DAVID to look for biological pathways and functional gene groups affected by maternal LCD. We identified significant regulation of five signalling pathways, including the valine, leucine and isoleucine degradation pathway, PPAR signalling pathway, glycerolipid metabolism pathway, sphingolipid metabolism pathway and fatty acid metabolism pathway (*P*<0.001, Table 2). Altogether, 31 GO terms, including fat metabolism related GO terms, such as fatty acid metabolic process, fat cell differentiation, were significantly changed (*P*<0.001, Table 3). Figure 5 shows the classification of biological processes in GO terms. All DEGs in the PPAR pathway are shown as Figure 6.

**Table 2:** The most enrichment pathways affected by maternal low chromium diet by using KEGG (*P*<0.001)

Pathway ID	Pathway name	Count	Fold enrichment	*P*-value
mmu00280	Valine, leucine and isoleucine degradation	12	16.44	6.45 x 10 ^-11^
mmu03320	PPAR signalling pathway	11	8.77	3.22 x 10 ^-7^
mmu00561	Glycerolipid metabolism	7	9.39	8.06 x 10 ^-5^
mmu00600	Sphingolipid metabolism	6	9.00	4.57 x 10 ^-4^
mmu00071	Fatty acid metabolism	6	8.40	6.32 x 10 ^-4^

**Table 3:** The most enrichment GO terms affected by maternal low chromium diet (*P*<0.001)

Term	Count	*P*-value	Fold enrichment	Catalogue
Fatty acid metabolic process	17	4.21 x 10 ^-11^	9.16	Biology process
Fat cell differentiation	9	1.54 x 10 ^-7^	14.63	Biology process
Brown fat cell differentiation	7	3.54 x 10 ^-7^	23.94	Biology process
Glycerolipid metabolic process	9	4.52 x 10 ^-5^	6.91	Biology process
Acylglycerol metabolic process	6	6.62 x 10 ^-5^	13.83	Biology process
Neutral lipid metabolic process	6	8.26 x 10 ^-5^	13.22	Biology process
Glycerol ether metabolic process	6	8.26 x 10 ^-5^	13.22	Biology process
Coenzyme metabolic process	9	9.37 x 10 ^-5^	6.24	Biology process
Organic ether metabolic process	6	1.13 x 10 ^-4^	12.39	Biology process
Response to insulin stimulus	6	3.03 x 10 ^-4^	10.08	Biology process
Regulation of hormone secretion	5	4.41 x 10 ^-4^	13.77	Biology process
TG metabolic process	5	4.41 x 10 ^-4^	13.77	Biology process
Cofactor metabolic process	9	4.87 x 10 ^-4^	4.90	Biology process
Regulation of fat cell differentiation	4	6.02 x 10 ^-4^	23.33	Biology process
Monocarboxylic acid transport	4	8.45 x 10 ^-4^	20.88	Biology process
Mitochondrial lumen	14	6.91 x 10 ^-9^	8.66	Cellular components
Mitochondrial matrix	14	6.91 x 10 ^-9^	8.66	Cellular components
Mitochondrial part	23	6.97 x 10 ^-9^	4.42	Cellular components
Mitochondrion	37	7.61 x 10-9	2.82	Cellular components
Monocarboxylic acid binding	7	1.55 x 10 ^-7^	27.34	Molecular function
Cofactor binding	14	2.01 x 10 ^-7^	6.53	Molecular function
Carboxylic acid binding	8	1.48 x 10 ^-5^	9.92	Molecular function
Coenzyme binding	10	1.97 x 10 ^-5^	6.59	Molecular function
Ligase activity, forming carbon–carbon bonds	4	2.77 x 10 ^-5^	60.26	Molecular function
Ligase activity, forming carbon–sulfur bonds	5	1.28 x 10 ^-4^	18.83	Molecular function
O-Acyltransferase activity	5	3.12 x 10 ^-4^	15.06	Molecular function
Acyl-CoA dehydrogenase activity	4	4.16 x 10 ^-4^	26.36	Molecular function
Acid-thiol ligase activity	4	5.02 x 10 ^-4^	24.81	Molecular function
Biotin binding	3	8.62 x 10 ^-4^	63.27	Molecular function
Fatty acid binding	4	9.55 x 10 ^-4^	20.08	Molecular function
Vitamin binding	7	9.78 x 10 ^-4^	6.10	Molecular function

**Figure 7:**
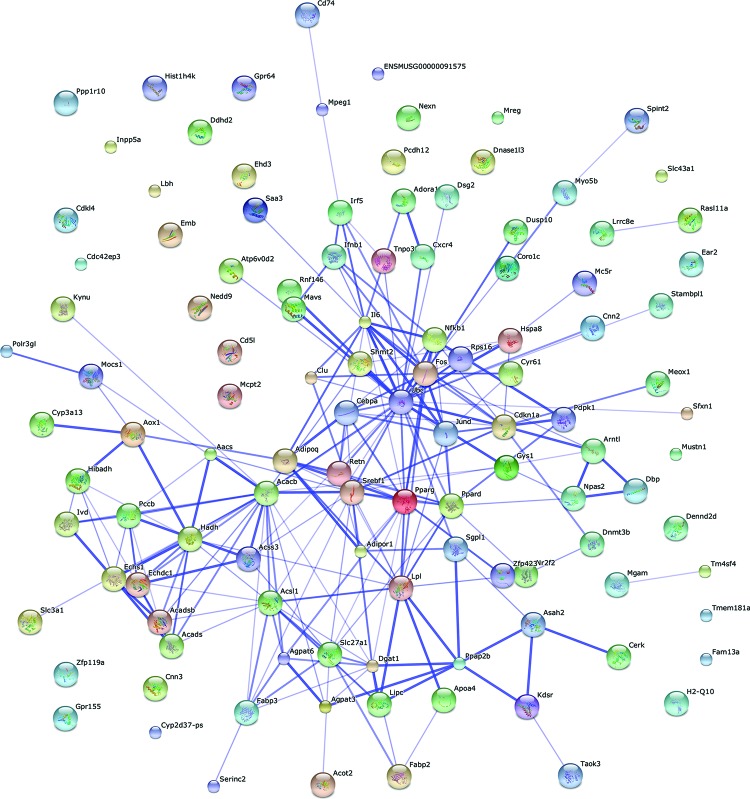
Gene interaction networks affected by maternal low chromium diet Interaction networks maps of DEGs. Thirteen nodes (more than 10 joint edges for each node) have total 190 joint edges, represents 88% of all the DEGs.

The 191 differentially expressed DEGs were mapped using String online software (confidence score =400). We identified that 86 nodes (genes) have 215 joint edges (interations) from these DEGs. In these nodes and edges, 13 nodes (each node with more than 10 joint edges) have 190 joint edges, representing 88% of all the nodes. These 13 nodes are ubiquitin C (*Ubc*), acetyl-coenzyme A carboxylase β (*Acacb*), FBJ osteosarcoma oncogene (*Fos*), *Srebf1, Pparg*, interleukin 6 (*Il6*), *Lpl*, acyl-CoA synthetase long-chain family member 1 (*Acsl1*), cyclin-dependent kinase inhibitor 1A (*Cdkn1a*), *Cebpa, Ppard*, diacylglycerol O-acyltransferase 1 (*Dgat1*) and hydroxyacyl-coenzyme A dehydrogenase (*Hadh*). They may have important functions in lipid metabolism affected by maternal low chromium in female offspring (Figure 7).

### Real-time PCR analysis

**Figure 8:**
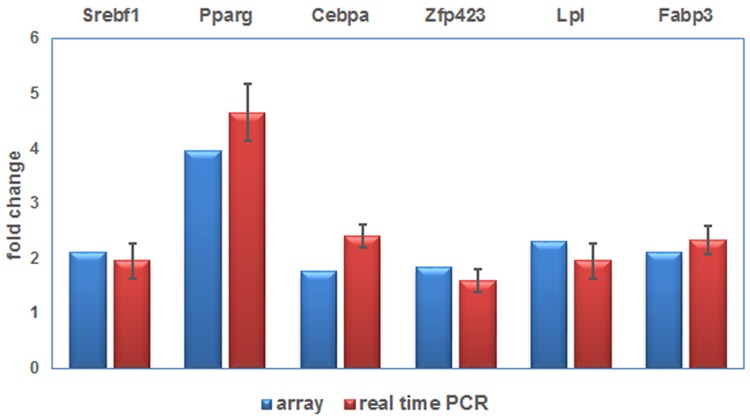
Real time PCR validation of differently expressed genes Real time PCR result of DEGs.

Because network analysis showed PPARγ pathway was in the centre of all 191 DEGs in LCD–CD group compared with CD–CD group, we selected six DEGs in PPARγ pathway to perform the real-time PCR analysis. The expression of *Srebf1, Lpl, Fabp3*,* Pparg, Cebpa* and *Zfp423* increased in the LCD–CD group (*P*<0.05). This result agreed with the corresponding data from the array (Figure 8).

## Discussion

In the present study, we found that the body weight of offspring from low chromium dams at birth and at 21 days did not differ from normal mice. However, at 32 weeks of age, the body weight of offspring from low chromium dams was greater than control mice. Kumar et al. [28] reported that the mice from vitamin B_12_-deficient mothers had reduced birth weights and increased body weights at 3 months. Additionally, maternal low chromium elevated fat-pad weight in pups at 8 months of age. A low-protein diet could increase adipocytes in offspring at 12 weeks of age [29]. Maternal Mg restriction increases body fat and decreases lean body and fat-free mass [30].

We also found that maternal low chromium increased the serum TG and LDL-C in pups at 8 months of age. Venu et al. [30] reported that the pups from Mg-restricted mothers had an increased plasma TG than control pups at 90 days. Maternal vitamin B_12_ restrictions increased TC (3 months and 12 months) and TG (3 months) in Wistar rat offspring [28]. Additionally, a 50% food restriction increased rat offspring serum TG [31].

Moreover, maternal low chromium increased serum leptin, TNF-α and IL-6 in the offspring at 8 months of age. A reverse diet could only correct the change in leptin and IL-6, but could not correct the change in TNF-α. Riddle et al. [32] found that intrauterine growth restriction (IUGR) increased circulating and adipose TNF-α in rat pups. Further, a maternal vitamin B_12_-restricted diet increased circulating and adipose tissue levels of TNF-α, leptin and IL-6 in offspring at 12 months of age [28].

Next, we performed microarray gene expression profile analysis to find the mechanism of the effect of a maternal low-chromium diet on the offspring. We found many members of the ZFP family were significantly up-regulated, such as *Zfp119a* and *Zfp423*. ZFP423 is an important transcription factor that activates adipogenic signalling. Yang et al. [33] found that obese mothers inhibited DNA methylation and histone modification of Zfp423 promoter, enhanced Zfp423 expression and activated adipogenic differentiation in foetal mice. Overexpressed Zfp423 leads to increase the expression of PPARγ and adipogenic commitment in progenitor cells. Downexpression of Zfp423 inhibits PPARγ expression and mitigates adipogenic differentiation [34].

By using gene pathway and network analysis, we identified that the PPAR pathway was significantly affected by maternal low chromium on the female offspring. Gene array and real-time PCR results also identified that maternal low chromium increased Pparγ, Srebp, Lpl, Fabp3 and Cebpa expression in the offspring adipose. Adipogenesis involves adipocyte differentiation, lipogenesis and lipid accumulation in adipose cells [35]. PPARγ and C/EBP are transcription factors, which can activate adipocyte differentiation [36]. In particular, C/EBPs can trigger adipogenic transcription factor PPARγ to induce the expression of lipogenic transcription factor SREBP1, activate Lpl and fatty acid synthase [37,38] and lead to adipocyte differentiation [36], intracellular TGs hydrolysis and fatty acid release from adipocytes [39].

**Figure 9:**
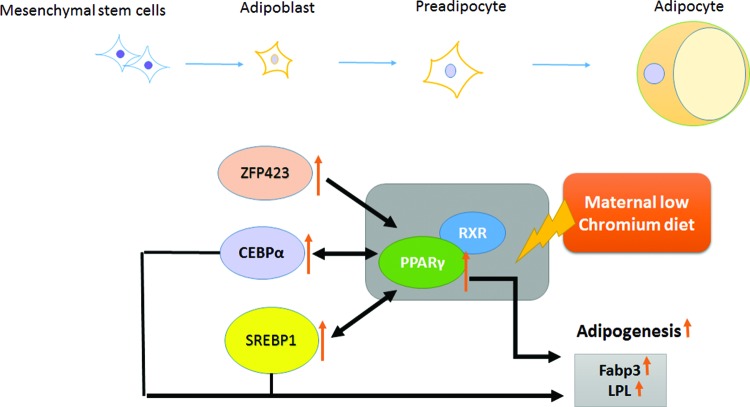
Schematic illustration of the effect of maternal low chromium on offspring through PPARγ pathway. PPARγ serves a key part in the regulation of adipocyte differentiation, adipogenesis and lipid metabolism, which are affected by maternal LCD in offspring adipose tissue. Maternal LCD triggers offspring adipose PPARγ pathway through activating ZFP423, CCAAT/enhancer-binding protein α (CEBPα) and SREBP1. Activated PPARγ binds to retinoid X receptor (RXR), enhancing its downstream target adipogenesis enzymes (Fabp3 and Lpl) and conversely inducing SREBP1 and CEBPα to increase circulation TGs.

In previous studies, obese rat and human subjects have enhanced PPARγ expression in the adipose tissue [40–42]. Furthermore, PPARγ expression is related to high-fat diet. In fasting status, PPARγ expression is inhibited [43]. In particular, PPARγ expression is inhibited in obese humans with a low-calorie diet [44] and in obese rats undergoing exercise [45,46]. Desai et al. [47] found that IUGR male offspring had increased PPARγ and its co-regulators protein expression at birth and the adult period in adipose. Ahmad et al. [48] reported that vitamin B_12_-deficient mothers have increased hepatic PPARγ expression in 12-month-old mice. Thus, our data supported that through activating ZFP423, CEBP and SREBP1, maternal low chromium triggers the PPARγ pathway, increased lipogenesis, resulting in lipid accumulation in adipose and increased circulating TGs level in offspring. The chromium supplement in standard rodent diet is enough to improve lipid metabolism status (Figure 9).

## Conclusions

In conclusion, our findings demonstrated that maternal LCD results in the potentially important alteration of adipose in the adult offspring mice. Furthermore, this change can not be reversed by normalizing the diet of the offspring. Our results mark a potential risk factor of maternal micronutrition in the incidence of obesity in offspring later in life. PPARγ serves a key part in the regulation of adipocyte differentiation, adipogenesis and lipid metabolism, which are affected by maternal LCD in offspring adipose tissue. Early intervention on PPARγ pathway maybe a potential strategy to modify the metabolic disturbance in offspring from low-chromium mothers.

## Abbreviations

AI: adiposity index
CD: control diet
C/EBP: CCAAT/enhancer-binding protein
Cebpa: CCAAT/enhancer-binding protein
DAVID: database for annotation, visualization and integrated discovery
DEG: differentially expressed genes
Fabp3: fatty acid binding protein 3
Gapdh: glyceraldehyde-3-phosphate dehydrogenase
LDL-C: low density lipoprotein-cholesterol
HbA1c: hemoglobin A1c
HDL-C: high density lipoprotein-cholesterol
GO: gene ontology
IL-1β: interleukelin-1 beta
IL-6: interleukelin-6
IUGR: intrauterine growth restriction
KEGG: Kyoto Encyclopedia of Genes and Genomes
LCD: low chromium diet
LDL: low-density lipoprotein
Lpl: lipoprotein lipase
PPAR: peroxisome proliferator activated receptor
Pparg: peroxisome proliferator activated receptor γ
Srebf1: sterol regulatory element binding transcription factor 1
TC: total cholesterol
TG: triglyceride
T2D: type 2 diabetes
TNF-α: tumor necrosis factor alpha
ZFP: zinc finger protein
*Zfp423*: zinc finger protein 423
